# Identification of five novel *SCN1A* variants

**DOI:** 10.3389/fnbeh.2023.1272748

**Published:** 2023-11-08

**Authors:** Baitao Zeng, Haoyi Zhang, Qing Lu, Qingzi Fu, Yang Yan, Wan Lu, Pengpeng Ma, Chuanxin Feng, Jiawei Qin, Laipeng Luo, Bicheng Yang, Yongyi Zou, Yanqiu Liu

**Affiliations:** ^1^Department of Medical Genetics, Jiangxi Maternal and Child Health Hospital, Nanchang, China; ^2^Jiangxi Provincial Key Laboratory of Birth Defect for Prevention and Control, Jiangxi Maternal and Child Health Hospital, Nanchang, China; ^3^School of Public Health, Nanchang University, Nanchang, China

**Keywords:** *SCN1A*, epilepsy, Dravet syndrome, *de novo*, seizures

## Abstract

**Background:**

Epilepsy is characterized by recurrent unprovoked seizures. Mutations in the voltage-gated sodium channel alpha subunit 1 (*SCN1A*) gene are the main monogenic cause of epilepsy. Type and location of variants make a huge difference in the severity of *SCN1A* disorder, ranging from the mild phenotype (genetic epilepsy with febrile seizures plus, GEFS+) to the severe phenotype (developmental and epileptic encephalopathies, DEEs). Dravet Syndrome (DS) is an infantile-onset DEE, characterized by drug-resistant epilepsy and temperature sensitivity or febrile seizures. Genetic test results reveal *SCN1A* variants are positive in 80% DS patients and DS is mainly caused by *de novo* variants.

**Methods:**

Trio-whole exome sequencing (WES) was used to detect variants which were associated with clinical phenotype of five probands with epilepsy or twitching. Then, Sanger sequencing was performed to validate the five novel *SCN1A* variants and segregation analysis. After analyzing the location of five *SCN1A* variants, the pathogenic potential was assessed.

**Results:**

In this study, we identified five novel *SCN1A* variants (c.4224G > C, c.3744_3752del, c.209del, c.5727_5734delTTTAAAACinsCTTAAAAAG and c.5776delT) as the causative variants. In the five novel *SCN1A* variants, four were *de novo* and the remaining one was inherited. All novel variants would be classified as “pathogenic” or “likely pathogenic.”

**Conclusion:**

The five novel *SCN1A* variants will enrich the *SCN1A* mutations database and provide the corresponding reference data for the further genetic counseling.

## 1. Introduction

The voltage-gated sodium channel alpha subunit 1 (*SCN1A*) gene encodes the α-subunit of a voltage-gated ion channel (NaV1.1), contributing to the initiation and propagation of action potentials and the regulation of neuronal excitability (Plumereau et al., 2022). As an archetypal channelopathy, the neuronal overexcitement leads to epilepsy characterized by recurrent unprovoked epileptic seizures (Scheffer and Nabbout, 2019; Plumereau et al., 2022). The phenotypic spectrum of epilepsy varies substantially across the patients harboring pathogenic variants in *SCN1A* gene (Zayat et al., 2022). The mild *SCN1A* disorder is genetic epilepsy with febrile seizures plus (GEFS+) (Fang et al., 2022). The severe form is developmental and epileptic encephalopathies (DEEs), including epilepsy with myoclonic atonic seizures (MAE), epilepsy of infancy with migrating focal seizures (EIMFS), and Dravet Syndrome (DS) (Scheffer and Nabbout, 2019; He et al., 2022). Phenotypic heterogeneity is commonly found in GEFS+ families (Grinton et al., 2022, p. 800; Türkyılmaz et al., 2022). GEFS+ patients tend to have self-limited and drug-reactive epilepsies (Scheffer and Nabbout, 2019) but normal cognitive development (Myers et al., 2018). DS is an infantile-onset DEE (Uchino et al., 2023), often accompanied by a fever or afebrile at initial seizure onset, subsequent motor and cognitive dysfunction, and intellectual disability in adults (Chilcott et al., 2022). Interestingly, DS patients harboring *SCN1A* variants 90% of which are *de novo* and only 5% of which are inherited account for 80% of cases (Kimura et al., 2005; Guerrini et al., 2010; Guerrini, 2012; Hirose et al., 2013).

The diagnosis of *SCN1A* disorders mainly depends on the clinical assessment and confirmation of diagnosis is based on genetic testing (Gil-Nagel et al., 2023). Moreover, using genetic testing to shorten the individual diagnosis time maybe benefit to select the antiseizure medications and improve the long-term quality of life in patients (Makiello et al., 2011; Wolff et al., 2019; Gil-Nagel et al., 2023; Matricardi et al., 2023). As a high-throughput and fast technique for genetic testing, Whole Exome Sequencing (WES) has been widely applied to identify *SCN1A* variants (Zhang et al., 2020; Gowda et al., 2023). To date, the Human Gene Mutation Database Gene Locus-Specific Database (HGMD) contains 2,584 *SCN1A* variants in total. Among these variants, truncations and missense changes account for the vast majority proportion (Zhou et al., 2021). Some research showed that truncations causing loss of function of Nav1.1 were associated with severe epilepsy, whereas missense variants often gave rise to mild phenotypes, which indicated the genotype–phenotype correlation (Zhou et al., 2021; Chen et al., 2022). However, the genotype–phenotype correlation in epilepsy patients with *SCN1A* variants is not definite enough (Chen et al., 2022).

Herein, taking advantage of Trio-WES, we detected five novel *SCN1A* variants associated with clinical phenotype in five probands with epilepsy or twitching, respectively. Then, the genetic source of five novel *SCN1A* variants were confirmed by the Sanger sequencing analysis. Simultaneously we analyzed their pathogenic potentials and locations. Following this, we performed prenatal diagnosis in pregnant women of family 1–3.

## 2. Materials and methods

### 2.1. Participants

Family members of five pedigrees were enrolled from the Jiangxi Maternal and Child health Hospital, Nanchang, China. General information and clinical manifestation were recorded, including gender, age, genetic relationship, and renal pathological phenomena. Peripheral blood was collected from all participants after signing written informed consent. The Clinical Research Ethics Committees of Jiangxi Maternal and Child health Hospital approved this study.

### 2.2. Trio-WES

Trio-WES of the proband and parents was used to detect variants which were associated with clinical phenotype. Genomic DNA was isolated from peripheral blood samples using a QIAamp DNA Mini Kit (Qiagen) and then was fragmented randomly by the ultrasonication (Covaris S220 Ultrasonicator). WES libraries were constructed, and the exons and adjacent splicing sites were amplified and sequenced on the high-throughput sequencing platforms (MGISEQ-2000, BGI) according to the manufacturer’s instructions. After generating the raw sequencing data from the sequencing platform, the sequencing adapters and low-quality sequences were trimmed. Taking advantage of BAW, all sequenced fragments were aligned and mapped to UCSC GRCh37/hg19 human reference genome and then processed for removing duplications and base quality score recalibration. Variant calling of single-nucleotide polymorphism (SNP) and indel (insertion or deletion) was carried out by GATK HaplotypeCaller. These variants were annotated and stratified for analysis using sunburst genetic analysis and interpretation platform.^1^ The *SCN1A* transcript of this study was NM_001165963.1.

Mutation nomenclature was based on the HGVS guidelines. These variants were filtered with multiple databases, such as population databases (dbSNP, 1,000 Genome, ExAC), disease databases (OMIM, HGMD, Clinvar) and biological information prediction tools (SIFT, Polyphen2, and Mutation Taster). The variants most significantly correlated with the clinical phenotypes were screened. Priority should be given to the variants which was found only in patients and do not exist in normal persons. The causative variants were classified according to the ACMG/ACG guidelines (Richards et al., 2015).

### 2.3. Identification of genotypes in five pedigrees

To validate the five novel variants and segregation analysis, adjacent regions variants in the *SCN1A* gene were amplified using the forward primer and the reverse primer designed by Primer-BLAST. Five specific primer pairs for detecting novel variants were listed in Table 1 and the PCR was performed through 2x Taq PCR Master MixII (KT211, TIANGEN). Amplification was carried out at 95°C for 5 min for initial denaturing, then 35 cycles at 95°C for 30 s, at 63°C (−0.5°C/cycles) for 30 s and at 72°C for 45 s, followed by a final extension of 8 min at 72°C in a T100 Thermal Cycler for the Classroom (BIO-RAD). The amplification products were sequenced by a sequencing provider (Tsingke, Changsha). The sequencing results alignment was completed by SeqMan Pro.

**Table 1:** Primers used to amplify the *SCN1A gene*.

Name	Sequence (5′-3′)	Used for the experiment of
SCN1A-E4-F	ACGCACAGTCTCCATCTTCTG	PCR for *SCN1A* Exon 4
SCN1A-E4-R	GGCTCTGACACCATCTCTGG	
SCN1A-E21-F	AAAGACCAGAGATTACTAGGGGA	PCR for *SCN1A* Exon 21
SCN1A-E21-R	TCACCCATCTGGGCTCATAAAC	
SCN1A-E22-F	TCCACCAATAGTCTTTCCCCTG	PCR for *SCN1A* Exon 22
SCN1A-E22-R	TTTCCCTACAAACTGCTGATGTG	
SCN1A-E26-F	GCCACAACCAAACAAACTCC	PCR for *SCN1A* Exon 26
SCN1A-E26-R	TTCCACAATTGGCTTTGTCA	
SCN1A-E29-F	CATGTACATCGCGGTCATCC	PCR for *SCN1A* Exon 29
SCN1A-E29-R	GGCTGTAAACAATTTGTCACCCA	

## 3. Results

### 3.1. Clinical characteristics and genetic analysis

**Figure 1:**
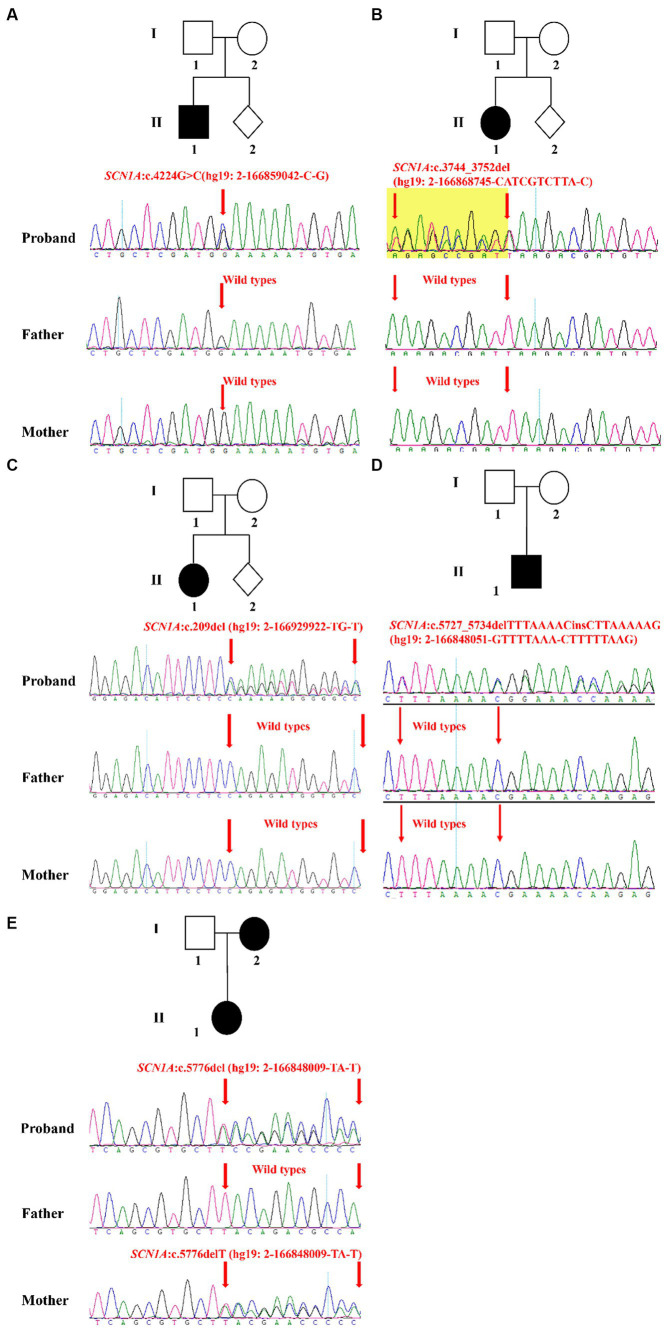
Validation and segregation analysis of *SCN1A* variants in five families. Upper: Pedigrees of family 1 **(A)**, family 2 **(B)**, family 3 **(C)**, family 4 **(D)**, and family 5 **(E)** with *SCN1A* variants; Lower: sequence chromatogram of the c.4224G > C **(A)**, c.3744_3752del **(B)**, c.209del **(C)**, c.5727_5734delTTTAAAACinsCTTAAAAAG **(D)** and c.5776delT **(E)** mutations on the *SCN1A* gene in the proband and his/her family members.

In this study, five families were recruited (Table 2). Epilepsy was found in all probands whose first seizure occurred before the age of 1 year, and incidence were often accompanied by psychomotor retardation or fever (Table 2). Through the treatment of antiseizure medications, the frequency and symptoms of epilepsy were gradually relieved (Table 2). In family 1, the proband II-1, male, was born with psychomotor retardation and epilepsy, while his parents were normal without clinical symptoms (Figure 1A; Table 2). In family 2, the female 6-month-old infant (proband II-1) was diagnosed as DS by clinic and had no family history (Figure 1B; Table 2). In family 3, the normal-appearing couple had an epileptic girl who suffered twitching once every half a month when she was born (Figure 1C; Table 2). In family 4, the normal-appearing couple had a recurrent epileptic (Figure 1D, Table 2). In family 5, clinical manifestations of the proband II-1 were twitching at the time of febrile illness (Figure 1E; Table 2). Her father was asymptomatic, while her mother had experienced the same symptoms in childhood, not now (Figure 1E; Table 2).

**Table 2:** Clinical phenotypes of five probands.

Case	Family 1: II-1	Family 2: II-1	Family 3: II-1	Family 4: II-1	Family 5: II-1
Sex	Male	Female	Female	Male	Female
Age (years)	6	2	14	10	4
Variant	c.4224G > C	c.3744_3752del	c.209del	c.5727_5734delTTTAAAACinsCTTAAAAAG	c.5776delT
Familial history	NO	NO	NO	NO	YES
Onset age	8 months	6 months	3 months	6 months	6 months
Onset frequency	Once per 0.5–1 month	Once per 0.5–1 year	Once per 0.5 months	Once per 10–20 days	Less
Fever	YES	NO	NO	YES	YES
Psychomotor development	Retardation	Retardation	Retardation	Normal	Normal
Magnetic resonance imaging	Normal	Not available	Normal	Normal	Normal
Clinical diagnosis	DS	DS	DS	DS	Not available
antiseizure medications response	Symptomatic relief	Symptomatic relief	Symptomatic relief	Symptomatic relief	Symptomatic relief

The result of Trio-WES confirmed maternity and paternity, and revealed that all patients harbored *SCN1A* heterozygous mutations. Subsequently, the Sanger sequencing analysis was performed to further validate these variants. In family 1, the c.4224G > C variant in the *SCN1A* gene existed in the proband and was absent in his healthy father and mother, which can explain the symptom of epilepsy (Figure 1A). In family 2, the proband carried one variant in the *SCN1A* gene (c.3744_3752del) associated with twitching, whereas it was not detected in parents (Figure 1B). In family 3 and 4, a definite diagnosis of epilepsy was obtained in the affected individual (I-1) by finding the c.209del variant and c.5727_5734delTTTAAAACinsCTTAAAAAG variant in the *SCN1A* gene, respectively (Figures 1C,D). In family 5, the detected variant in the *SCN1A* gene (c.5776delT) of the proband inherited from her mother (Figure 1E). Moreover, the proband and her mother were sick with the similar symptom.

### 3.2. Analysis of variants

**Figure 2:**
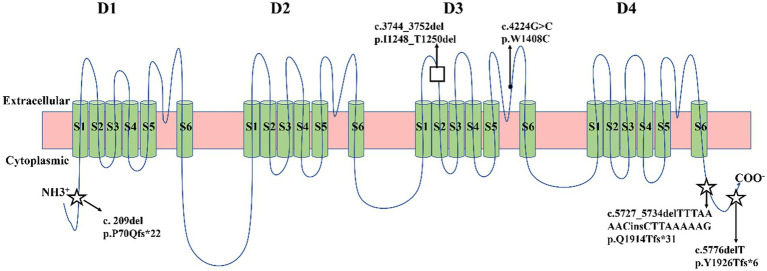
Schematic location of five variants in the transmembrane topology of a voltage-gated sodium channel encoded by *SCN1A* gene. Five green circles represented α-helical transmembrane segments (S1-S6), which can combine with a pore loop to form a homologous domain (D1-D4). Squares represented missense variants. Black dots represented in-frame deletion variants. Five-pointed stars represented frameshift variants.

Five identified variants were not recorded in HGMD and Clinvar databases in this study (Table 3). In addition, these novel variants had never been reported in previous research, two of which were located in the C-terminal domain (c.5727_5734delTTTAAAACinsCTTAAAAAG/p. Gln1914Thrfs*31, c.5776delT/p. Tyr1926Thrfs*6), one in the pore loop connecting segment 5 (S5) and segment 6 (S6) of domains 3 (D3) (c.4224G > C/p.Trp1408Cys), one in D3S1- S2(c.3744_3752del/p. Ile1248_Thr1250del), and one in N-terminal domain (c.209del/p. Pro70Glnfs*22) (Figure 2). All novel variants were not present in the population frequency databases (Table 3). After bioinformatics-based prediction, the structure or function of protein may be disrupted by these variants. Following the ACMG guideline (Richards et al., 2015), all novel variants would be classified as “pathogenic” or “likely pathogenic” (Table 3).

**Table 3:** Novel mutations identified in five families.

Variant	c.4224G > C	c.3744_3752del	c.209del	c.5727_5734delTTTAAAACinsCTTAAAAAG	c.5776delT
Amino acid change	Trp1408Cys	Ile1248_Thr1250del	Pro70Glnfs*22	Gln1914Thrfs*31	Tyr1926Thrfs*6
Variant type	Missense	Deletion	Frameshift	Frameshift	Frameshift
Population frequency	–	–	–	–	–
HGMD	–	–	–	–	–
Clinvar	–	–	–	–	–
Literatures	–	–	–	–	–
SIFT	0(damaging)	–	–	–	–
PolyPhen	1(Probably_damaging)	–	–	–	–
REVEL	0.96(damaging)	–	–	–	–
ACMG criteria	PS2 + PM2 + PP3	PS2 + PM2 + PM4	PVS1 + PS2 + PM2	PVS1 + PM2	PVS1 + PS2 + PM2

## 4. Discussion

On clinical, genetic testing should be carried out in an infant presenting with generalized convulsions status epilepticus or recurrent febrile seizure. The most common monogenic cause of epilepsy is *SCN1A* variants (Brunklaus et al., 2022). Genetic test results reveal *SCN1A* variants were positive in 80% DS patients and 10% GEFS+ patients (Scheffer and Berkovic, 2000). Therefore, *SCN1A* genetic testing is mainly used for confirming the clinical diagnosis of DS and should be discouraged in GEFS+ patients (Hirose et al., 2013). In this study, five patients were born with epilepsy, twitching or febrile illness. Suspecting DS, we performed genetic testing and detected five novel *SCN1A* variants, respectively.

In the five variants, four were *de novo* and the remaining one was inherited. Previous studies reported that *de novo* accounted for 90% of *SCN1A* variants in DS patients, only 5% inherited variants (Kimura et al., 2005; Guerrini et al., 2010; Guerrini, 2012; Hirose et al., 2013). Milder GEFS+ phenotypes were observed in the family members harboring inherited variants (Hirose et al., 2013). Consistent with that reported in previous studies, the majority of detected variants were *de novo* in this study and the mother with inherited variants of family 5 experienced relatively mild symptoms in childhood which have disappeared now. In addition, the phenotypes varies substantially across the patients having *de novo* pathogenic variants in *SCN1A* gene, which is related to type and location of variants (de Lange et al., 2018).

Functional studies have shown missense mutations in the ion-pore and voltage-sensor regions, resulting in a lack of sodium current, caused serious clinical symptoms (Zuberi et al., 2011). Here, the patient of family 1 had psychomotor retardation and epilepsy, which were serious clinical symptoms caused by the c.4224G > C variant in the pore loop. This novel missense mutation was located at the 1408th amino acid residue where a deleterious nonsense mutation (c.4223G > A) had been reported before (Fujiwara et al., 2003, p. 1). The DS patient with c.4223G > A showed similar symptoms such as severe mental decline and childhood epilepsies (Fujiwara et al., 2003, p. 1). Here, the novel in-frame deletion (c.3744_3752del) variant in D3S1- S2 was detected in a DS patient. A recorded in-frame deletion variant (c.3740_3751del) was included in this mutation region (Wang et al., 2022). The c.3740_3751del variant was *de novo* and resulted in DS, according to *SCN1A* mutations database (Wang et al., 2022). Comparing with frameshift mutants causing premature truncation, in-frame deletion mutants lead to the loss of one or more amino acids of proteins and preserve function. However, electrophysiological analysis indicated that the *SCN1A* in-frame deletions will also exhibit complete loss-of-function (Wang et al., 2022). A newly discovered frameshift variant (c.209del) was in N-terminal domain. The clinical phenotype of this variant was the worst in five novel *SCN1A* variants. Surprisingly, in this study the patients harbored c.5727_5734delTTTAAAACinsCTTAAAAAG or c.5776del mutants in the C-terminal domain, suffered from the different severities of epilepsy and febrile convulsions. Frameshift mutants behind the two mutants (c.5741_5742delAA and c.5788delC) were both *de novo* and associated with DS (Harkin et al., 2007; Zuberi et al., 2011). Though the pathogenicity mechanism was haploinsufficiency for Nav1.1 (Harkin et al., 2007; Zuberi et al., 2011), the phenotypes of C-terminal variants were different, which increased the difficulty for genotype–phenotype prediction.

**Figure 3:**
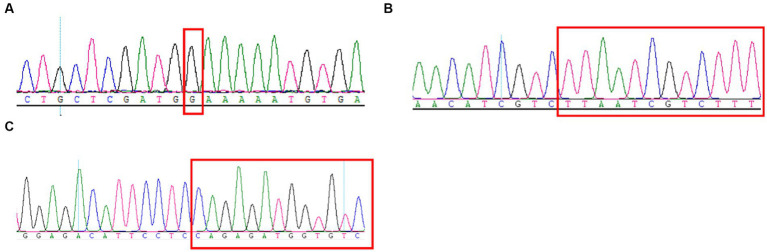
Sanger sequencing results of the c.4224G > C **(A)**, c.3744_3752del **(B)**, and c.209del **(C)** mutations on the *SCN1A* gene in the fetuses of family 1–3.

Here, the mothers of family 1–3 were both pregnant again. They expressed a desire to undergo prenatal diagnosis for causative variants detection (c.4224G > C, c.3744_3752del and c.209del) in fetuses to prompt the birth risk of infants with the similar symptom of probands. The sanger sequencing results showed the likely pathogenic variant (c.4224G > C and c.3744_3752del) were not found in the fetus of family 1 and 2 (Figures 3A–C). In addition, no pathogenic variant (c.209del) was detected in the fetus of family 3 (Figure 3C). Finally, three couples were both willing to continue with the pregnancy and the three infants displayed normal phenotype.

More often than not, *de novo* mutations occurring either in post zygotically or a single gamete are a one-off event and the risk of *de novo* mutations recurrence is 1% ~ 2% (Alison et al., 2023). Meanwhile, *de novo* mutations are constantly produced in both somatic and germ cells to form gonadal mosaicism during growth and development, which can significantly increase the recurrence risk (Acuna-Hidalgo et al., 2016). Depienne et al. observed that *de novo* mutations in the *SCN1A* gene remained a small possibility with associated recurrence risk (Depienne et al., 2006). A follow-up study showed that the percentage of mutant cells in the gonadal mosaicism was positively related to the severity of the phenotype (Depienne et al., 2010). Xu et al. found that 8.6% parents of DS children were *SCN1A* gene mutation mosaicism (Xu et al., 2015), which create a higher risk for family reproduction. Thus, we recommended that prenatal diagnosis was made for pregnant women of family 1–3. Sanger sequencing can detect some parental mosaicism (Xu et al., 2015), but a degree of misdetection rate still exists. Provided that a personalized risk assessment and full disclosure of all potential risk is essential for prenatal diagnosis.

In conclusion, we performed gene diagnosis for five families with epilepsy or twitching by WES-trio, respectively. As a result, five novel variants in *SCN1A* gene were identified, confirmed, and analyzed. These variants will enrich the *SCN1A* mutations database and provide the corresponding reference data for the further genetic counseling or genotype–phenotype correlations.

## Data availability statement

The datasets presented in this study can be found in online repositories. The names of the repository/repositories and accession number(s) can be found in the article/supplementary material.

## Ethics statement

The studies involving humans were approved by the Clinical Research Ethics Committees of Jiangxi Maternal and Child health Hospital. The studies were conducted in accordance with the local legislation and institutional requirements. Written informed consent for participation in this study was provided by the participants’ legal guardians/next of kin. Written informed consent was obtained from the individual(s), and minor(s)’ legal guardian/next of kin, for the publication of any potentially identifiable images or data included in this article.

## Author contributions

BZ: Data curation, Formal analysis, Writing – original draft. HZ: Data curation, Formal analysis, Writing – original draft. QL: Investigation, Writing – original draft. QF: Investigation, Writing – original draft. YY: Investigation, Writing – original draft. WL: Investigation, Writing – original draft. PM: Investigation, Writing – original draft. CF: Investigation, Writing – original draft. JQ: Investigation, Writing – original draft. LL: Investigation, Writing – original draft. BY: Project administration, Supervision, Writing – review & editing. YZ: Funding acquisition, Project administration, Writing – review & editing. YL: Project administration, Writing – review & editing.

## Footnotes

^1^ https://genetics.bgidx.cn/
